# Effects of energy drink major bioactive compounds on the performance of young adults in fitness and cognitive tests: a randomized controlled trial

**DOI:** 10.1186/s12970-014-0044-9

**Published:** 2014-11-07

**Authors:** Maximiliano Kammerer, Jaime A Jaramillo, Adriana García, Juan C Calderón, Luis H Valbuena

**Affiliations:** Indeportes Antioquia and School of Nutrition and Dietetics, University of Antioquia, UdeA, Calle 70 No 52-21, Medellín, Colombia; Colcafé S.A.S, Medellín, Colombia; Indeportes Antioquia, Medellín, Colombia; Physiology and Biochemistry Research Group-Physis, Department of Physiology and Biochemistry, Faculty of Medicine, University of Antioquia, Medellín, Colombia; Exercise Physiology Laboratory from Indeportes Antioquia and Physiology and Biochemistry Research Group-Physis, University of Antioquia, Medellín, Colombia

**Keywords:** Energy drink, Caffeine, Taurine, Physical condition, Cognitive ability

## Abstract

**Background:**

The consumption of beverages containing caffeine and taurine before exercising has been associated with increased physical and psychological performances and has been promoted to support the emotional state and provide vitality to consumers. However, there are contradictory results on these issues, it is not clear the effect of every major compound in relation to the whole effect of the beverages and there is a lack in knowledge about their degree of safety for consumption.

**Methods:**

This study used a double-blind, placebo controlled, randomized, crossover design. Fourteen male volunteer soldiers from the Colombian army performed different tests to measure their cardiorespiratory fitness (VO_2_max and maximum heart rate), time to exhaustion, strength (isometric strength), power (vertical jump), concentration (Grid test) and memory (Digits test) after drinking 250 ml of one of the following beverages: one with 80 mg caffeine, one with 1000 mg taurine, one with 80 mg caffeine plus 1000 mg taurine, a commercial energy drink (Red Bull®) or a placebo drink. Subjects were caffeine-consumers that avoided caffeine during the day of evaluation. All beverages were matched in flavor and other organoleptic properties to the commercial one, were bottled in dark plastic bottles and were administered in identical conditions to the participants. Differences between treatments were assessed using repeated measures and analysis of variance.

**Results:**

The mean ± SD values of VO_2_max, maximum heart rate, time to exhaustion, right handgrip strength, left handgrip strength, vertical jump, Grid test and Digits test were 61.3 ± 6.2 ml/kg.min, 196 ± 6.8 beats per min, 17 ± 1.2 min, 56.8 ± 6.6 kgf, 53.1 ± 5.9 kgf, 41.1 ± 3.8 cm, 19.9 ± 5.9 observed digits and 10.9 ± 3.1 remembered digits after drinking a placebo drink. Comparisons among the commercial drink, caffeine, taurine, caffeine plus taurine and placebo treatments did not show statistically differences in the results of the performed tests. No adverse effects were reported by the participants.

**Conclusion:**

The consumption of caffeine (80 mg) and taurine (1000 mg) or their combination does not increase the physical and cognitive ability in young adults during exercise.

## Background

Energy drinks are soft drinks, generally carbonated, composed of different ingredients such as caffeine, taurine, glucuronolactone, carbohydrates and vitamins with different rates of absorption to which the proposed effects of these beverages are attributed [1-3]. Furthermore, depending on the brand, these drinks have other ingredients such as amino acids, minerals and vegetable extracts, together with acidulants, preservatives, flavorings and colorings. These types of products have gained popularity worldwide since the 1990s, increasing exponentially the consumption among adolescents, athletes, and even senior citizens [4]. Manufacturers of these products promote their consumption with statements offering a variety of benefits among which increased physical performance, improved reaction rate, increased attention, higher concentration, improved emotional state and weight loss are included [4]. These are desirable characteristics for anyone, especially for active individuals. The interaction of the major bioactive compounds has been proposed as responsible for the alleged effects of these products; however, such statements have not been well studied and are not fully supported. Furthermore, there are contradictory results on the issue. Some studies attribute the alleged effects in improving the state of concentration and physical endurance to the combination of the compounds that make up these products [5,6]. Other studies point out that is very likely that most of the observed effects after consumption of these drinks are mainly produced by caffeine [7-9]. On the contrary, other studies have found no increase in the performance after consumption of these drinks [10].

Currently, many people seek benefits for their physical and mental health situation that has led them to use these types of drinks; nonetheless, despite an increased consumption by the general population, there is a lack of knowledge about the physiological effects of the compounds used in the formulations, the level of security in their consumption and their position within food standards. This is probably because there are few well-designed studies that provide accurate and conclusive findings on the subject.

Therefore, in this study we evaluated the efficacy and safety of energy drink consumption in a randomized, controlled, double-blinded, crossover trial and thus we were able to assess the direct involvement of caffeine and taurine, as major bioactive compounds of energy drinks, on physical and cognitive condition in young adults.

## Methods

### Subjects

A sample of 9 or 13 subjects was calculated in order to find differences in cognitive or physical tests. This was done by using PRIMER software 3.02 (PRIMER-E Ltd, United Kingdom), after establishing a confidence level of 95% and a power of 80%. Nonetheless, 14 volunteered male soldiers, members of the National Army of Colombia Seventh Division, were included in the study. No cigarette smokers or users of psychoactive substances, or subjects under any medication (e.g. beta blockers) were included. People who consumed more than 600 ml of coffee or more than 5 units of colas per day were excluded [11].

All participants underwent a complete physical examination and an electrocardiogram to determine their physical and mental conditions and all were diagnosed as healthy. In addition, they all signed the informed consent before the physical and cognitive tests. The procedures performed were endorsed and approved by the Indeportes Antioquia Research Committee and the National Army Research Committee in order to provide protection to the study participants.

### Experimental design

This research used a double-blind, placebo controlled, randomized, crossover design, in which each participant visited six times the Exercise Physiology Laboratory from Indeportes Antioquia, in Medellín, Colombia, at 1540 m above sea level. Three fitness tests and two cognitive condition tests were applied in each visit. All evaluation sessions were separated between 48 or 72 hours and three subjects were evaluated per day. In the first session the subjects performed the tests, without drink consumption, to become familiar with their development. Testing was always conducted at the same time (2 pm-5 pm) in an environment with controlled temperature and humidity of 25°C and 60%, respectively. A training specialist and a psychologist with extensive experience in sports fields were responsible for measuring all variables.

Participants maintained their regular food intake, workplaces and recreational activities during study participation, but did not train at high intensity eight hours before the tests, did not eat heavy meals two hours before the tests and avoided caffeine consumption during the day of evaluation. Since the participants were soldiers, they had a very controlled schedule regarding food and exercise every day. So we are sure that during the day of evaluation the variation in these variables was kept to the minimum. All subjects used appropriate clothing for physical exercise.

Before developing the tests all subjects were randomly assigned (with the help of Excel 2007, Microsoft Co, USA) to a treatment (a placebo drink, a caffeinated drink, a caffeine and taurine combined drink, a drink with only taurine, or a commercial energy drink) and after 45 minutes performed each of the tests. All subjects received all beverages during the study. The composition of the five beverages is illustrated in Table 1.

**Table 1:** **Beverages composition per serving (250 ml) used in the study**

**Drink number**	**Drink**	**Caffeine (mg)**	**Taurine (mg)**
1	Placebo	0	0
2	Caffeinated drink	80	0
3	Drink with caffeine and taurine	80	1000
4	Drink with taurine	0	1000
5	Commercial drink (Red Bull®)	80	1000

The first four beverages were developed with a flavor profile similar to the commercial energy drink brand (Red Bull®). For the design and formulation of each treatment, final contents of other ingredients such as sweeteners, acidulants, stabilizers, and colorings were determined apart from the major bioactive components, to ensure a close level to the physicochemical properties such as acidity, pH, refractometric degrees, color, smell and sensory profile similar to the commercial energy drink.

Carbonated-based water was used for all drinks and the appropriate powder mixture, previously formulated, was added. All bioactive and additional ingredients were selected from certificated suppliers and were of food grade.

Brix (Mettler Toledo RE50 refractometer), pH and acidity (Mettler Toledo Seven Easy pH meter), and color (Hunter Lab D25LT colorimeter) were analyzed in all beverages.

In order to ensure similarity between all beverages, and keep the double-blind design of the study, organoleptic properties such as smell, color, sweetness, acidity, body and overall impression were evaluated. This analysis was supported by the Colcafé expert panel (Medellín, Colombia). Decisions on formulation adjustments were based on the results obtained and analyzed with FIZZ Sensory Analysis Software (France).

All treatment beverages, including the commercial one, were pasteurized at 80°C for 15 seconds, bottled in 250 ml polyethylene terephthalate bottles, identified with numeric codes and administered in identical conditions to the participants. The day of the experiment, each participant consumed all 250 ml of the assigned drink in a period of 10 to 15 minutes.

### Evaluations

#### Food consumption

A 24-hour recall and an analysis of the complete menu provided by the Army to the soldiers were used to determine the dietary intake of the subjects in the study. Portion sizes, brands and cooking methods were recorded. We used the official food composition table from University of Antioquia’s School of Nutrition and Dietetics (Medellín, Colombia) to determine the calories and nutrients consumed by the tested subjects and the average values were used in the analysis of consumption.

#### Level of physical activity

The level of physical activity was measured using the Global Physical Activity Questionnaire (GPAQ) of the World Health Organization [12]. Subjects were interviewed to remember the number of days from the last week when they did vigorous intensity or moderate intensity activities in three aspects (work or home activities, travel to different places or leisure activities) and the number of hours and minutes per day of different activities performed. This multidomain questionnaire was used since it was validated in both developed and developing countries and several populations including Latino people [13-15]. It is now used worldwide and this fact enables comparisons across diverse populations.

### Physical tests

#### Determination of cardiorespiratory fitness

All participants completed a set of ergospirometry tests that allowed us directly monitoring their cardiorespiratory behavior. An open circuit spirometry (Oxycon Delta, Jaeger, Spain) previously calibrated was used. A ramp protocol was used in test performing [16]. After explaining the test to each subject, the resting ergospirometry parameters were taken and the test was started with a three minutes warming up period at a speed of 3.5 mile/h with a band inclination of 1%, then the speed was increased by 0.5 mile/h every minute until exhaustion. To measure participant recovery, subjects continued walking with an intensity and duration similar to the warming up period for three minutes, followed by other two minutes of passive recovery in standing position. After the test each subject was disconnected from the computer and the information obtained was recorded. Maximum oxygen consumption (VO_2_max), maximum heart rate (HRmax) and time to exhaustion (TTE) were determined from test results.

#### Strength calculation

A static dynamometry test of the upper limbs was performed. We used handgrip strength, which has been considered a simple, fast and reliable test for measuring human strength in different populations [17-20]. For handgrip strength measurements (finger flexor force), a previously calibrated Takei Kiki Kogyo digital dynamometer (Japan), with a transducer 1270A, series 86002, 90 kg, was used. Each of the participants performed a ten minutes guided warming up, focusing on the muscle groups that were evaluated. Subsequently, the evaluated subject stood up and held the transducer, in such a way that the upper end of this rests on the palm, under thenar and hypothenar regions, and the lower end on the second to the fifth finger second phalanx. Then, with the upper limb away from the body and no support points for the transducer, the soldier proceeded to perform bilateral maximum handgrip strength for five seconds; this procedure was repeated after one minute of recovery and the highest value was recorded [21].

#### Power calculation

Three vertical jumps were performed on a contact, previously calibrated, platform (Newtest Power timer series 10327) that calculated the flight time. Data obtained allowed to calculate the height reached and the subject mechanical power. Functioning of the test was explained to participants; then, there was a warming up emphasizing the muscles of the lower limbs. After subjects performed three nonconsecutive free jumps using arms, the height was measured by taking into account the flight time. Out of the three jumps performed the highest flight time was recorded [22].

### Cognitive tests

#### Focused attention test (Grid)

This instrument allowed the evaluation of outside attention, both broad and narrow according to basic principles of attention control [23]. Its execution consisted of pointing out (according to the instructions provided previously) the numbers 1 to 38 that appeared in a grid with different sized digits, sequentially.

#### Digit span WAIS subtest (Digits)

Attention, resistance to distraction and immediate auditory memory were measured with a memory function subtest part of Wechsler Adult Intelligence Scale (WAIS) test. It consists of asking subjects to repeat two sets of numbers, which were previously read in direct and reverse order, respectively. A level of nine numbers in direct order and eight numbers in reverse order was used for the test. For this study we used the digit span test by changing the numbers in each sample to avoid learning, but keeping the series, times and original guidelines [24].

These are simple, of short-duration tests, which evaluate cognitive capacities important during the practice of different sports. Both tests have been previously used in similarly designed approaches to the research question of the present work (see [1]).

### Statistical analysis

We checked the assumptions of normality, homogeneity and randomness of the different variables (VO_2_max, HRmax, TTE, right and left handgrip strength -RHS and LHS-, vertical jump, concentration and memory). The differences between treatments were measured using a 5x5 table (treatments *vs.* sessions) of repeated measures analysis of variance (ANOVA). The lower significant difference was developed by post-hoc analysis with the Tukey test. All data is reported in the text and the tables as mean ± SD. Minitab 16.0 software was used to perform the analysis. A value of p < 0.05 was considered statistically significant.

## Results

The 14 participants had an age of 20 ± 1 years (mean ± SD), a weight of 68 ± 7.2 Kg and a height of 170.7 ± 5.4 cm. The total energy consumed by the soldiers was 2654 ± 97 Kcal/day and the percentage of it provided by carbohydrates, proteins and fat was 59%, 14% and 27%, respectively. Total hours spent in physical activity were also calculated and multiplied by the metabolic equivalent (MET) minutes per week to determine an energy expenditure of 4536 ± 123 MET/min/week. Table 2 shows further characterization of the physical activity of the population.

**Table 2:** **Level of physical activity of the soldiers involved in the study**

**Variable**	**Value**
Domestic work activities (min/day)	60 (15–129)*
Movement to different places (min/day)	52.5 (30–60)*
Movement activities	12 participants
No movement activities	2 participants
Recreational activities (min/day)	25.7 (15–40)*
Sedentary behavior (min/day)	60 (60–90)*

^*^ *Since these data were not normally distributed (Shapiro-Wilk test), medians and (interquartile ranges) are shown.*

The Table 3 presents the descriptive statistics of the physiological variables measured in the soldiers during the study. No significant differences were found in the values of VO_2_max (p = 0.736), in HRmax (p = 0.715), TTE (p = 0.836), strength (RHS p = 0.096 and LHS p = 0.607), power (VJ) (p = 0.066), concentration tests (Grid, p = 0.763) and immediate memory (Digits, p = 0.168).

**Table 3:** Descriptive statistics for the variables evaluated and the five treatments in all participants (n = 14)*

	**Placebo**	**Caffeine**	**C + T**	**Taurine**	**Commercial drink**	**P value**
**VO**_**2**_ **max (ml/kg/min)**	61.3 ± 6.2	61.1 ± 5.2	61.8 ± 5.4	60.6 ± 5.1	61.1 ± 5.1	0.736
**HRmax (beats per min)**	196 ± 6.8	196.5 ± 4.5	197.1 ± 6	197.8 ± 6.6	196.4 ± 6.2	0.715
**TTE (min)**	17 ± 1.2	17 ± 1.03	16.8 ± 1.2	16.9 ± 1.4	16.8 ± 1.2	0.836
**RHS (kgf)**	56.8 ± 6.6	55.3 ± 6.7	57.5 ± 6.2	55.4 ± 6.9	56.2 ± 5.8	0.096
**LHS (kgf)**	53.1 ± 5.9	53.5 ± 4.9	54 ± 6.3	52.6 ± 7	52.9 ± 5.5	0.607
**VJ (cm)**	41.1 ± 3.8	41.7 ± 3.8	41.5 ± 4.2	42.5 ± 4.4	40.9 ± 4.1	0.066
**GRID (number of observed digits)**	19.9 ± 5.9	19.8 ± 4.4	20.6 ± 4.5	19.1 ± 4.7	19.3 ± 3.5	0.763
**DIGITS (number of remembered digits)**	10.9 ± 3.1	10.6 ± 2.9	11.0 ± 2.8	11.2 ± 2.7	10.3 ± 3.1	0.168

*Values are mean ± SD. C + T: caffeine + taurine, VO_*2*_ *max: maximal oxygen uptake, HR: heart rate, TTE: time to exhaustion, RHS: right handgrip strength, LRH: left handgrip strength, VJ: vertical jump, GRID: concentration test, DIGITS: immediate memory test.*

No adverse effects were reported by the subjects after drinking the beverages and during the time they were at the laboratory.

Our results suggest that there are not statistically significant differences among the results of the commercial drink, drinks with different bioactive compounds and placebo in the various tests carried out by the soldiers, at the delivered dose with a confidence level of 95%.

## Discussion

Our results indicate that the consumption of caffeine (80 mg) and taurine (1000 mg) in an energy drink before performing cognitive and fitness test has no effects on cardiorespiratory fitness indices (VO_2_max, HRmax, TTE), strength (RHS and LHS), power (VJ), concentration (Grid) and immediate memory (Digits).

We want to point out that all beverages were prepared and evaluated, previously to be administered to the soldiers, by an expert group of people with the help of industrial up-to-date technology and chemicals. So, their lack of effect on the studied variables cannot be proposed to result from inadequate quality of the beverages. Also, we want to make clear that the aim of the study was not to evaluate the effect of increasing doses of caffeine or taurine on the physical and cognitive performance, rather, it was to evaluate the effects of the amounts of these compounds currently found in most energy drinks available for free consumption.

Some of our findings contrast with those of others [1,6,25,26]. The effects have been attributed to the combination of ingredients like caffeine, taurine, glucuronolactone and B vitamins in these drinks. However, we must clarify that in those studies the main ingredients of an energy drink (caffeine and taurine) were not isolated and the drinks were separated as control (placebo) and experimental (other drinks) groups without, in several cases, good masking of the treatments. In our study each subject was his own control which can minimize the bias introduced when evaluating compounds such as caffeine and taurine, since the individual response to the consumption of these substances may be different. Also, our methodology allowed a good masking of the treatments and gives strength to this study. On the other hand, our findings are consistent with the results obtained by Candow and colleagues [10], Umana-Alvarado and Moncada-Jiménez [27], and Nelson and colleagues [28] in which the consumption of the commercial beverage had not positively influenced the outcome of the physical tests performed. It is important to note that in our study we assessed the cardiorespiratory fitness directly (through ergospirometry) which can provide more reliable information than when tests are performed indirectly through a stress test.

Results shown in Table 3 demonstrate that the consumption of the commercial energy drink did not affect the HRmax of the evaluated subjects, in agreement also with the findings of Baum and Weiss [29], Bichler and coworkers [30] and the recent work of Nelson and colleagues [28]. In contrast, in Geiß and colleagues’ work [6], lower HR values were reported after endurance athletes consumed a commercial energy drink. However, the subjects in the last research were athletes, while our study was conducted in a different population (soldiers). Since the soldiers studied have very similar dietary patterns, physical activity, employment and lifestyle (hours of sleep, free time management, level of education), the differentiating effect that could have these patterns on fitness and cognitive tests can be minimized.

In a recent report with similar design to ours, Nelson and colleagues [28] did not find any change in ride time-to-exhaustion and some cardiovascular performance variables on a sample of similar size of young, moderately fit subjects, after they drunk a caffeinated and taurinated commercial drink. Since we used subjects with higher VO_2_max values and the results were the same, it is tempting to speculate that the lack of effects of the studied compounds may persist in people within a relatively large range of physical capabilities.

On the other hand, a paper showed a significant increase in VO_2_max, TTE and maximal workload in a stress test after consuming a taurine supplement [31]. The authors postulated that this could be due to attenuation of exercise-induced damage to deoxyribonucleic acid and increased exercise capacity by its protective properties at cellular level, which differs with the results found in this research. Unfortunately, we did not further explore in our subjects the mechanisms mentioned by that study [31].

In this research it was not found that the bioactive compounds have a positive effect on immediate memory and concentration tests, as opposite to several reports [1,5,32,33], where the effect was attributed to either the caffeine or an ingredient combination. In those studies, however, each energy drink main compound was not evaluated. In our case, no differences were found in cognitive tests when combined or isolated bioactive compounds were consumed; possibly because either the amount of caffeine used is insufficient to positively influence the concentration and immediate memory test outcomes or there is not an actual effect. These results are consistent with findings made by others [34,35], who found no effects in the subject memory and alertness and concentration state. The dose we administered to the subjects was between the range of 1–2 mg/kg proposed by some authors to have physiological effects on several cognitive and physical functions when administered in energy drinks [3,36-38]. Although some authors have proposed that low doses of caffeine are associated with increased energy, alertness and vigilance, motivation and concentration, but not with memory in non-athletes [37], i.e., there is a task specific profile of response to caffeine, the variability in results obtained in active people with energy drinks suggests that this is not clear yet (see for instance [1,5,32 and results from the present work]). Further investigations are necessary for people who exercise to identify the lower caffeine doses that produce beneficial effects in their performance that can really be worthwhile [39-41], to evaluate the effect on several variables of cognitive performance more sensitive to low doses of caffeine [37] and also to study the potential additive effect of the components of energy drinks. The history of caffeine consumption and withdrawal in the subjects studied is not expected to affect our results, since in both low (about 50 mg/day) and high caffeine (about 200 mg/day) consumers, a period of withdrawal comparable to that used in the present work has no effect on the cognitive responses measured after a caffeine challenge [37,42]. The lack of influence of the consumer type on different variables affected or not by caffeine, has also been reported in works which involved people with a history of caffeine consumption ranging from zero to more than 200 mg/day [26]. So, it is likely that caffeine habituation (from zero to 200 mg/day) is not an important variable to explain results from studies evaluating the effect of energy drinks on sports performance.

It has also been suggested that taurine can help in skeletal muscle contractile function, by increasing the sarcoplasmic reticulum Ca^2+^ content and muscle force generation [43-46]. Moreover, the potential synergistic or additive effects of caffeine and taurine when administered in energy drinks deserve attention [3]. In this study, however, neither the drink with taurine, nor the taurine combined with caffeine nor the commercial drink favored a higher generation of strength than the placebo drink.

## Conclusions

The different bioactive compounds studied here at the concentrations (caffeine 80 mg and taurine 1000 mg per serving of 250 ml) found in most currently available energy drinks do not have positive effects on cardiorespiratory fitness test (VO_2_max, HRmax and TTE), force or power; neither on concentration test nor on immediate memory in young, healthy, active adults. Results from the combination of beverage ingredients also showed no difference from the results obtained when consuming a placebo drink. No adverse effects were recorded after ingestion of the studied beverages.

## Abbreviations

Digits: digit span WAIS subtest
Grid: focused attention test
GPAQ: Global Physical Activity Questionnaire
HRmax: maximum heart rate
LHS: left handgrip strength
MET: metabolic equivalent
RHS: right handgrip strength
TTE: time to exhaustion
VJ: vertical jump
VO_2_max: maximum oxygen consumption
WAIS: Wechsler Adult Intelligence Scale
